# Anticipating protein evolution with successor sequence predictor

**DOI:** 10.1186/s13321-025-00971-z

**Published:** 2025-03-21

**Authors:** Rayyan Tariq Khan, Pavel Kohout, Milos Musil, Monika Rosinska, Jiri Damborsky, Stanislav Mazurenko, David Bednar

**Affiliations:** 1 Loschmidt Laboratories, Department of Experimental Biology and RECETOX, Faculty of Science, Masaryk University, Brno, Czech Republic; 2 International Clinical Research Center, St. Anne’s University Hospital Brno, Brno, Czech Republic; 3 Department of Information Systems, Faculty of Information Technology, Brno University of Technology, Brno, Czech Republic

**Keywords:** Protein design, Activity, Adaptation, Evolution, Thermostability, Solubility, Evolutionary trajectory

## Abstract

**Abstract:**

The quest to predict and understand protein evolution has been hindered by limitations on both the theoretical and the experimental fronts. Most existing theoretical models of evolution are descriptive, rather than predictive, leaving the final modifications in the hands of researchers. Existing experimental techniques to help probe the evolutionary sequence space of proteins, such as directed evolution, are resource-intensive and require specialised skills. We present the successor sequence predictor (SSP) as an innovative solution. Successor sequence predictor is an in silico protein design method that mimics laboratory-based protein evolution by reconstructing a protein's evolutionary history and suggesting future amino acid substitutions based on trends observed in that history through carefully selected physicochemical descriptors. This approach enhances specialised proteins by predicting mutations that improve desired properties, such as thermostability, activity, and solubility. Successor Sequence Predictor can thus be used as a general protein engineering tool to develop practically useful proteins. The code of the Successor Sequence Predictor is provided at https://github.com/loschmidt/successor-sequence-predictor, and the design of mutations will be also possible via an easy-to-use web server https://loschmidt.chemi.muni.cz/fireprotasr/.

**Scientific Contribution:**

The Successor Sequence Predictor advances protein evolution prediction at the amino acid level by integrating ancestral sequence reconstruction with a novel in silico approach that models evolutionary trends through selected physicochemical descriptors. Unlike prior work, SSP can forecast future amino acid substitutions that enhance protein properties such as thermostability, activity, and solubility. This method reduces reliance on resource-intensive directed evolution techniques while providing a generalizable, predictive tool for protein engineering.

**Graphical abstract:**

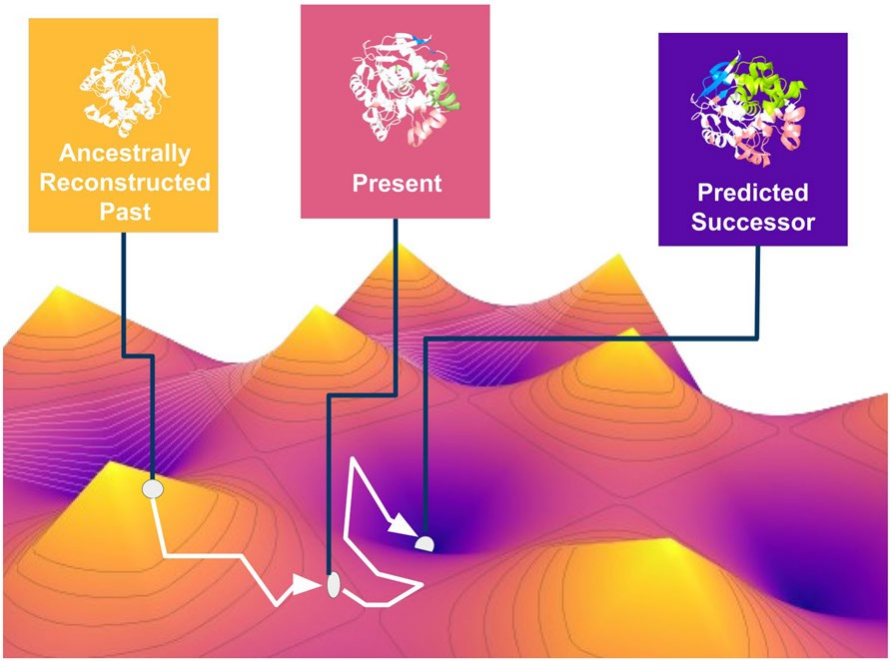

**Supplementary Information:**

The online version contains supplementary material available at 10.1186/s13321-025-00971-z.

## Introduction

Evolution is a general term that describes the changes in inherited traits of biological entities through successive generations, generally in response to environmental changes [1]. While it can be modelled or described at many levels of biological organisation and varying levels of accuracy, for this study, we will focus on protein evolution.

Protein evolution can be reduced to two key steps: amino acid mutation and the fixation of the mutated protein in a population [2, 3]. An individual mutation may result from errors in DNA replication during cell division, exposure to mutagens, or a viral infection. The probability of fixation of this new mutation in the population depends on the fitness effect of the mutation itself. The new variant can be neutral, deleterious, or beneficial. While this two-step model is useful, it is only descriptive and not predictive [4]. For this reason, it cannot be used to predict upcoming mutations in the future and their fixation probability [5]. Thus, generally the field of evolutionary predictions has been limited to forecasting adaptive processes, as opposed to amino acid level mutations. Efforts to improve these kinds of predictions are typically focused on the aspect of selection. This neglects the fact that adaptive processes are reliant on new mutations, which in turn do have predictable biases [6]. Yet most evolutionary predictions are focused on evolution of infectious diseases, cancer and or other somatic evolutions at the phenotypic level [7]. An in silico methodology that can predict evolution at the amino acid level can ease our reliance on cost prohibitive methodologies such as those in the realm of directed evolution [8].

Directed evolution refers to experimental techniques used to engineer a protein and possibly understand the effect of various mutations on a protein and their fixation probabilities. These techniques allow a user to probe a protein’s evolutionary space. They are used to improve protein characteristics and, sometimes, even to confer new characteristics onto a protein [9] by selecting or screening many variants. The markers for improvement in protein characteristics due to induced mutations can be taken as a proxy for fixation probabilities of the induced mutation in a natural environment if it occurs without human intervention. While this model has not been framed in such a way previously, it closely models the concepts of classic Darwinian/positive selection [10].

However, directed evolution experimental techniques require specialised skills and are both time and resource-intensive. Thus, any in silico technique for predicting and mimicking laboratory-based protein evolution would be of great use for the design of proteins with novel properties. As of this writing, we have only come across one technique, Proseeker, which uses physicochemical characteristics and structure to pick sequences that have higher probabilities of evolving a desired function [11]. However, the technique was designed specifically for binding proteins. It uses smaller peptide sequences (13 amino acids), and it does not filter AAindices, i.e., physiochemical descriptors [12], rather it uses all available AAindices. This leaves room for refinement by selection of more useful indices.

On the other hand, ancestral sequence reconstruction (ASR) complements these approaches by leveraging phylogenetic trees and sequence alignments to trace evolutionary changes and infer ancestral protein sequences [13–15]. By reconstructing evolutionary histories, ASR reveals positions in protein sequences where selective pressures have driven adaptations. Building on this foundation, Combinatorial Libraries of Ancestors for Directed Evolution (CLADE) was developed to target specific positions identified through ASR [16, 17]. CLADE leverages the uncertainty inherent in ancestral reconstructions by creating combinatorial libraries, focusing on positions with the highest uncertainty for mutagenesis. This strategy enables the exploration of sequence space at evolutionarily significant sites, yielding superior results compared to consensus mutagenesis, which targets conserved residues from sequence alignments [18]. However ASR only lets us explore the evolutionary past of a sequence. Combining evolutionary insights with physicochemical properties through AAindices holds great potential for predicting evolutionary successors that align with physical evolutionary pressures.

To this end, we propose a novel method called Successor Sequence Predictor (SSP), which can mimic laboratory-based protein evolution. It reconstructs the evolutionary history of a protein sequence and then suggests amino acid substitutions based on trends observed in the evolutionary history of the protein when projected through the lens of various, carefully selected, physicochemical descriptors. Introducing the predicted mutations would enhance specific protein properties. For example, if SSP is used on a protein that in the history of its evolution was experiencing a selection pressure towards becoming more thermostable, the predicted substitutions will most likely make the mutant protein even more thermostable, and likewise for other physicochemical properties of the protein. We describe the method in detail and then conduct its critical validation against five different experimental data sets targeting properties such as thermostability, activity, and solubility. A dataset of amino acid sites that were determined to be positively selected by various evolutionary sequence analysis methodologies was also incorporated in the validation [19].

## Materials and methods

### Selection of AAindices

**Fig. 1:**
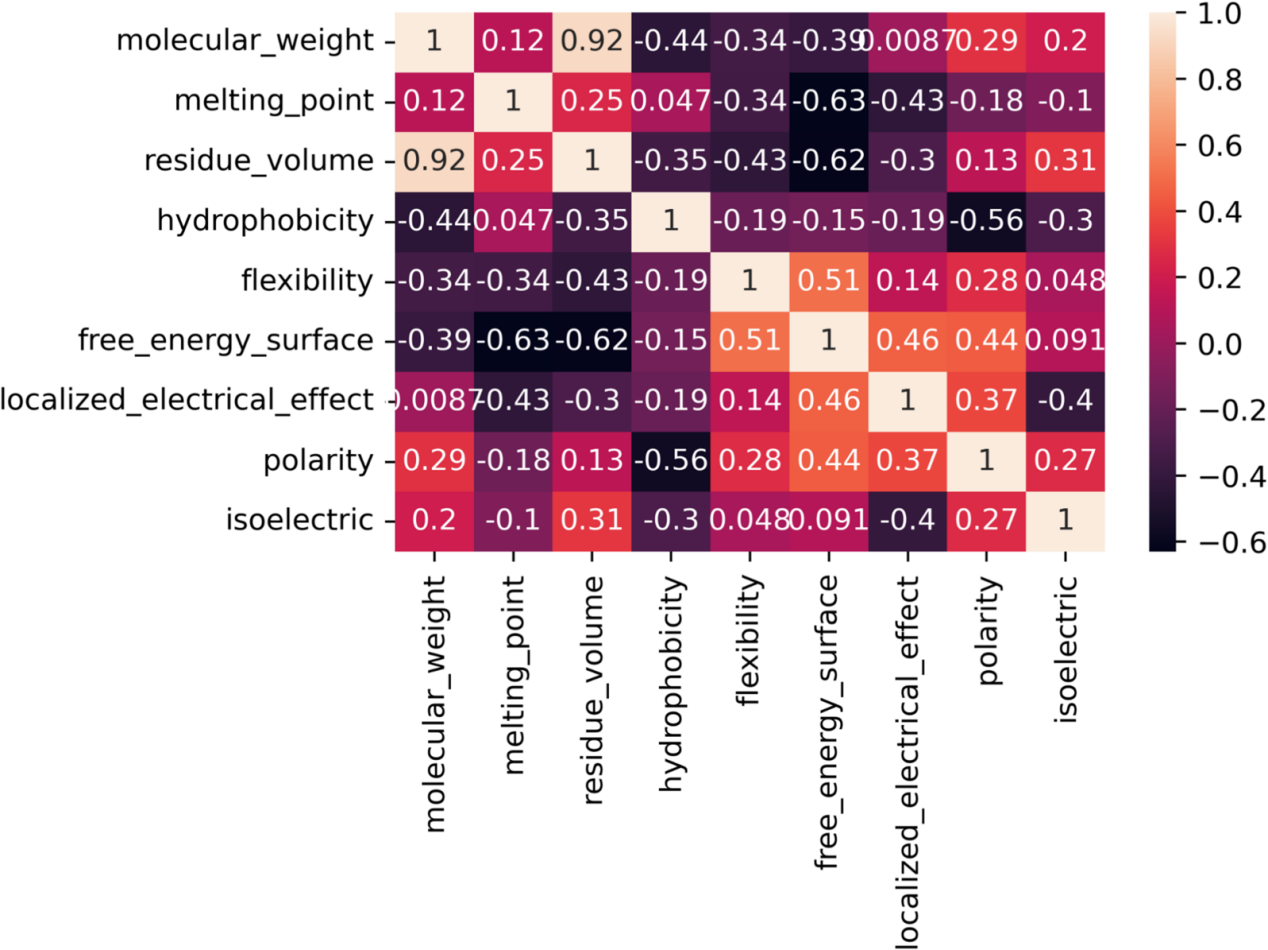
Pearson correlation matrix of selected AAindices. The correlation coefficients are colour-coded from dark purple at − 0.7 to off-white at 1.0. The indices are summarised in Table 1

Nine AAindices were manually selected after consideration, to reflect a variety of possibly relevant physiochemical descriptors (Table 1). While the AAindex stores many more indices, they were considered inappropriate due to factors such as redundancy or context-specific physiochemical descriptions. Correlation analysis ensured that the nine selected indices had significant differences (Fig. 1), and while molecular weight and residue volume indices were similar, they were retained due to the slight nuances of how they evaluated different amino acids. Thus no indices were discarded.

**Table 1:** The AAindices used to analyse amino acid evolution

Index	Property	Reference
FASG760101	Molecular weight	[20]
FASG760102	Melting point	[20]
GOLD730102	Residue volume	[21]
WOLR790101	Hydrophobicity index	[22]
BHAR880101	Average flexibility indices	[23]
BULH740101	Transfer free energy to the surface	[24]
FAUJ880108	Localised electrical effect	[25]
ZIMJ680103	Polarity	[26]
ZIMJ680104	Isoelectric point	[26]

The correlations among the individual indices are presented in Fig. 1

### Successor sequence predictor workflow

**Fig. 2:**
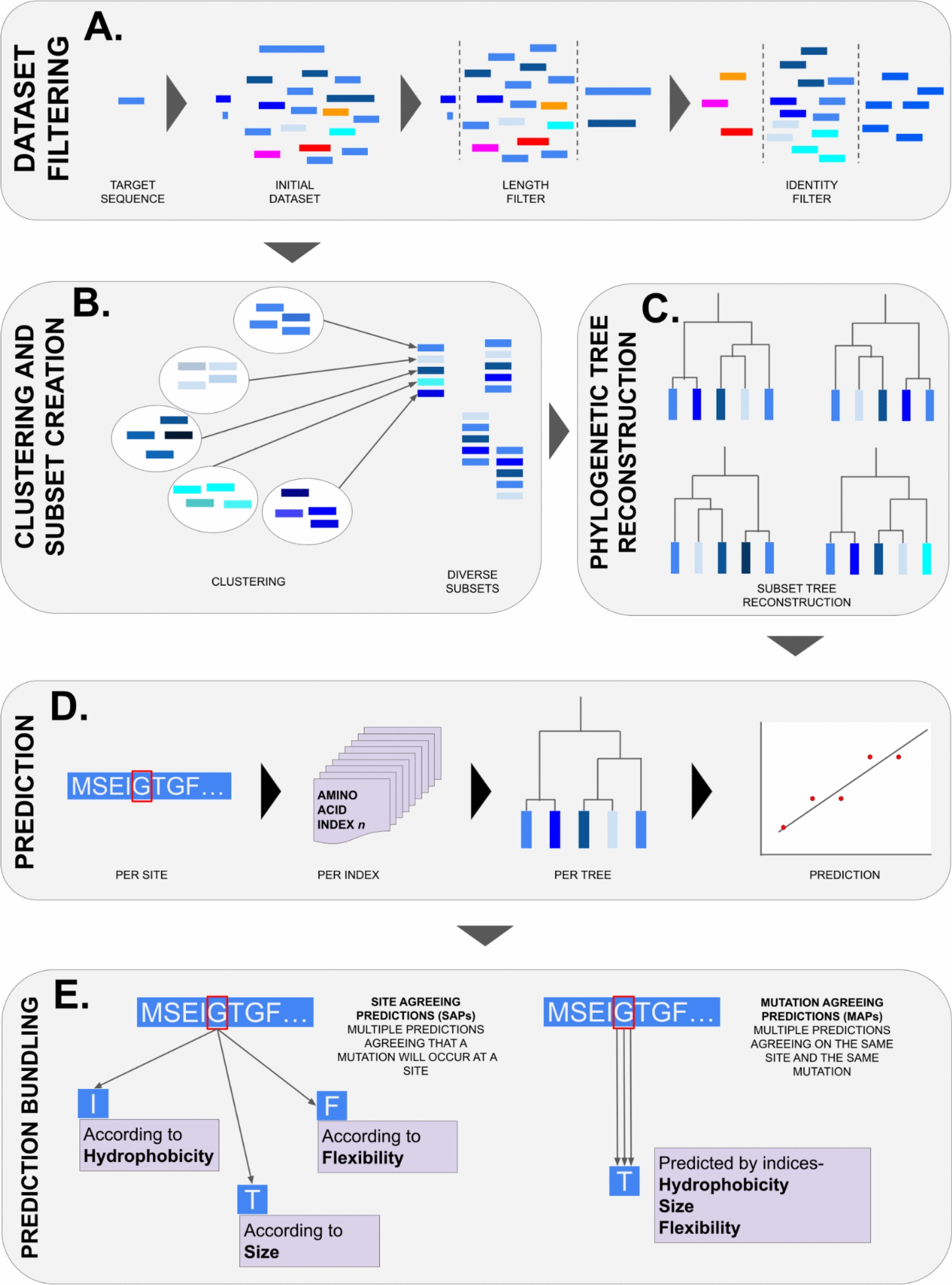
A generalised overview of the Successor Sequence Predictor (SSP). **A** Initial curation and filtering of the target protein’s dataset. **B** Further division of data using a clustering methodology. **C** Phylogenetic tree reconstruction and ancestral sequence reconstruction for the nodes on the trees. **D** Trend construction and amino acid prediction. **E** Prediction bundling

Successor Sequence Predictor follows the workflow outlined in Fig. 2. Firstly, the FASTA sequence of a target protein is used to identify a dataset of homologous sequences using BLAST [27]. Only sequences with 30–90% sequence identity to the target are retained. A length filter is then applied to keep sequences within 80–120% of the target protein’s length. The remaining sequences are clustered using USEARCH at 90% sequence identity, and one sequence from each cluster is randomly selected (Fig. 2A).

The dataset obtained from these steps is divided to construct multiple phylogenetic trees, each containing 150 sequences (Fig. 2B) with the final number of phylogenetic trees dependent on the dataset size. Before processing, the dataset is amended to ensure sequence headers do not contain problematic special characters (e.g., parentheses, colons, semicolons, or numbers at the start of headers) that could disrupt the function of the utilised tools. Next, sequences are clustered based on similarity using SigClust [28] with the parameter c = 150, producing up to 150 clusters. Sequence files are then generated with the following rules:

1. Each target sequence must appear in at least one sequence file.
2. One sequence is randomly selected from each cluster for each file.
3. To maximize diversity, the algorithm avoids reusing sequences from clusters unless all options are exhausted.
4. Every sequence file must contain the target sequence.

Once the sequence files are prepared, ClustalOmega [29] creates a multiple sequence alignment (MSA) for each file.

The MSAs are then processed using the standard FireProt^ASR^ workflow [14]. RAxML [30] is employed to construct a phylogenetic tree for each MSA using the maximum-likelihood algorithm. RAxML runs in its SSE3 version, using 50 bootstraps and the best-fit evolution matrix suggested by IQ-TREE. Once the calculation is completed, the minimum ancestral deviation algorithm is used to root the generated trees (Fig. 2C) [31]. This approach generally leads to highly similar trees across multiple runs, yet a level of stochasticity can still be expected as most of the employed tools rely on heuristic algorithms.

Rooted phylogenetic trees and corresponding MSAs are loaded into LAZARUS [32] to calculate posterior probabilities for each sequence file. LAZARUS uses the “codeml” module, the appropriate evolutionary matrix, and a fixed branch lengths, with gap reconstruction disabled (this step is handled by the gap correction algorithm implemented in FireProt^ASR^). Based on the posterior probabilities and predicted ancestral gaps, ancestral sequences are reconstructed for each node in the phylogenetic tree. The main path from the root to the target sequence is identified for each phylogenetic tree (Fig. 2D).

The sequences from the target and all ancestral nodes to the root are extracted into a separate file and aligned using ClustalOmega. Finally, a Python script employing the “numpy” and “sklearn.linear_model” libraries [33] predicts the successor sequence as the next step along a regression curve, following these steps (Fig. 2E):

1. For each column in the MSA (referred to as a "Trajectory"), a matrix of amino acid physicochemical features is generated, with each column representing one of nine selected AAindices.
2. For each column, a vector of changes in physicochemical features is calculated, weighted by the evolutionary distance from the root node.
3. This vector is used to train a linear regression model to predict the next amino acid in the trajectory, mimicking laboratory-based protein evolution.
4. The distance between consecutive amino acids in the trajectory (based on AAindex values) is calculated as the average distance between nodes along the main path in the phylogenetic tree.
5. Separate regressions for each physicochemical feature are aggregated to assign categories and bundle predictions (Table 2).
6. This process is repeated for every column in the MSA and each sequence file.

**Table 2:** An example of the generalised prediction bundling scheme for three different levels of prediction: prediction, site agreeing prediction (SAP), and mutation agreeing prediction (MAP)

Type	Amino acid	Position	Prediction	Index
Prediction	A	12	L	Size
Site agreeing prediction (SAP)	A	12	L	Size
			R	Hydrophobicity
Mutation agreeing prediction (MAP)	A	12	L	Size
				Polarity
				Flexibility

Several precautions are taken to minimize over-interpretation of the linear regression approach. The regression plot is normalized by the number of transitions (amino acid substitutions) in the trajectory. If an amino acid remains unchanged across successive ancestors at a given position, it is treated as part of a group and not penalized in scoring. Transitions between groups are counted only when they occur.

Key metrics include the penultimate transition, which flags any changes inconsistent with the overall trend as a "*break trend*." Trajectory *sequentiality* is scored out of 100, with a perfect score achieved when each transition in a positive trend increases the feature's value compared to the previous one. *Fluctuations* in a trajectory are measured by dividing the number of different amino acids by the number of amino acid groups, reflecting the trajectory's stability or variability. Sites with fewer than three transitions are excluded from predictions.

These scores are used to rank successor amino acid predictions for each site, index, and phylogenetic tree. The highest-ranking predictions are those with high *sequentiality*, high *fluctuation*, and no *break trend* at the penultimate amino acid position. Each amino acid prediction is averaged across sites and trees.

When multiple predictions agree at a specific site but differ in the mutation type, they are referred to as Site Agreeing Predictions (SAPs). Conversely, when predictions from different AAindices align on the same mutation, they are consolidated into a single prediction, known as a Mutation Agreeing Prediction (MAP) (Table 2).

### Validation datasets

Mutational datasets investigating a physicochemical property of any specific protein were searched through the literature. The ones with large enough datasets, which also had enough overlap with predicted mutations (thus allowing us to validate them,) were selected. This includes homolog sets for levoglucosan kinase—UniProt ID B3VI55 [34], cold shock protein CspB—UniProt ID P32081 [35], ADP-ribosylarginine hydrolase—Uniprot ID P54922 [19], and aminoglycoside 3'-phosphotransferase—UniProt IS P00552 [36].

Individual datasets were compiled in different ways. The levoglucosan kinase set was found via the *in-house* SoluProtMut^DB^ database [37] by searching for a protein with a large number of experimentally validated single-point mutations and their effects on the solubility of the protein. Similarly, the cold shock protein CspB dataset was found in the *in-house* FireProt^DB^ database [38], by searching for a protein with a large number of experimentally validated single-point mutations and their effects on the thermostability of the protein. In cases where multiple values were available for a single mutation, the mean was taken. ADP-ribosylarginine hydrolase dataset was picked as it was one of the example cases for Slodkowicz and Goldman’s online tool [19] for Structure Integrated with Positive Selection. ADP-ribosylarginine hydrolase was picked after a literature review, due to the sheer number of single-point mutations tested (fully site saturated) on the target protein by Melnikov et al. [36]. This naturally presented a perfect test case for SSP. Individual and detailed dataset handling steps are noted in SI 2.

## Results

### Dataset statistics

**Fig. 3:**
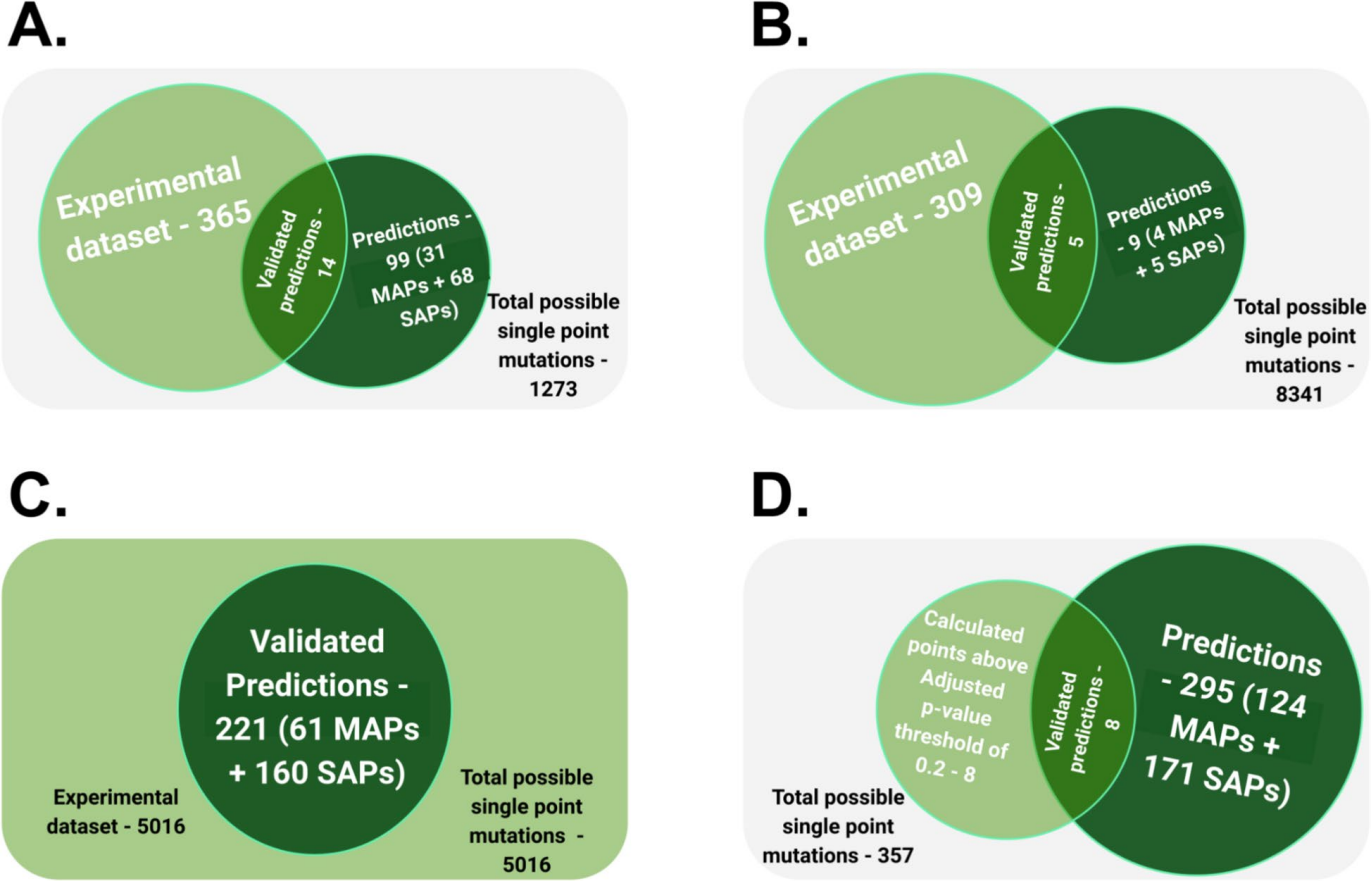
The visualisation of overlaps between the available experimental data and the predicted data. **A** Overlap metrics for Cold shock protein CspB set (FireProt^DB^ dataset—[38], **B** Overlap metrics for levoglucosan kinase set [34] **C** Overlap metrics for Aminoglycoside 3'-phosphotransferase set [36], and **D** Overlap metrics for ADP-ribosylarginine hydrolase set [19]. The experimental data are represented by a light green circle, while a dark green circle represents predicted data

We tested the performance of SSP on the homolog sets for levoglucosan kinase (solubility), cold shock protein CspB (thermostability), ADP-ribosylarginine hydrolase (selectivity), and aminoglycoside 3ʹ-phosphotransferase (activity). It is important to note that with the exception of Aminoglycoside 3ʹ-phosphotransferase dataset, none of the other datasets used in the study have the values for the relevant effect for every possible point mutation that SSP predicts. Thus it is not possible to validate all predictions made by SSP. The results section only shows validation based on all mutational data points that SSP predicted and for which experimental labels were available. Figure 3 summarises the total single-point mutational space, the available experimental values, the number of predictions, and the overlaps between the two.

### Engineering thermostability

SSP predictions for Cold shock protein CspB were compared to experimental data points with known effects of the mutation on protein thermostability from a collated dataset stored in the database FireProt^DB^ [38]. In cases where values from multiple datasets were available, the mean values were noted. E3Q was the only MAP that was supported by more than three indices. E3K was supported by 2 indices, and all others were SAPs. The results are provided in Table 3.

**Table 3:** Effects of mutations generated by SSP on the thermostability of cold shock protein validated against the collated FireProt^DB^ dataset [38]

Mutation by SSP	Mean ΔΔG (kcal/mol)	△Tm (°C)^a^	Prediction agreement type
L2R	+ 0.4	N/A	Site agreeing
E3K	− 2.48	+ 16.6	Mutation agreeing
E3Q	− 1.09	+ 7.3	Mutation agreeing
E3R	− 1.65	+ 16.0	Site agreeing
E3V	− 1.8	N/A	Site agreeing
D24N	+ 0.66	− 6.9	Site agreeing
A46E	+ 0.07	− 5.0	Site agreeing
A46K	− 1.41	+ 8.4	Site agreeing
A46L	− 0.8	N/A	Site agreeing
E50K	+ 0.33	− 5.6	Site agreeing
N55D	− 0.46	+ 3.9	Site agreeing
N55K	0	+ 0.8	Mutation agreeing
N55S	+ 0.2	N/A	Site agreeing
E66K	− 2.17	+ 12.9	Site agreeing

^a^*N/A* data not available

There were 365 total mutations in the FireProt^DB^ dataset [38], of which 18% were enhancing mutations in terms of thermostability (ΔΔG lower than − 1 kcal/mol), 55% were neutral (ΔΔG from − 1 kcal/mol to 1 kcal/mol), the remaining 27% were destabilising (ΔΔG greater than 1 kcal/mol). The thresholds for stabilising, neutral and destabilising categories were taken from the FireProt^DB^.

From the 14 mutations predicted by SSP, six were stabilising. The other eight mutations had ΔΔG values between − 1 kcal/mol and 1 kcal/mol and can thus be classified as neutral. Seven out of ten mutations also increased the melting temperature (*T*_m_) of the protein, and three were destabilising (Table 3).

### Engineering solubility

SSP predictions for levoglucosan kinase were compared to experimental data from Klesmith et al. [34] available in the SoluProtMutDB [37]. This comparison assessed how well the SSP predictions matched the known effects of mutations on protein solubility.The mutations I3L and I3F (supported by two different indices) had a neutral effect on solubility. Both mutations predicted by SSP, D9G and K38Q, are known to have a slightly enhancing effect on solubility. Only V200A showed a slightly negative effect on solubility in *E. coli* (Table 4)*.* This suggests that the expressed mutants produced via SSP do not compromise their solubility.

**Table 4:** Effects of mutations generated by SSP on the solubility of levoglucosan kinase [34]

Mutation by SSP	Effect on solubility	Prediction agreement type
I3L	Neutral	Mutation agreeing
I3F	Neutral	Mutation agreeing
D9G	Positive	Site agreeing
K38Q	Positive	Site agreeing
V200A	Negative	Site agreeing

### Engineering activity

**Fig. 4:**
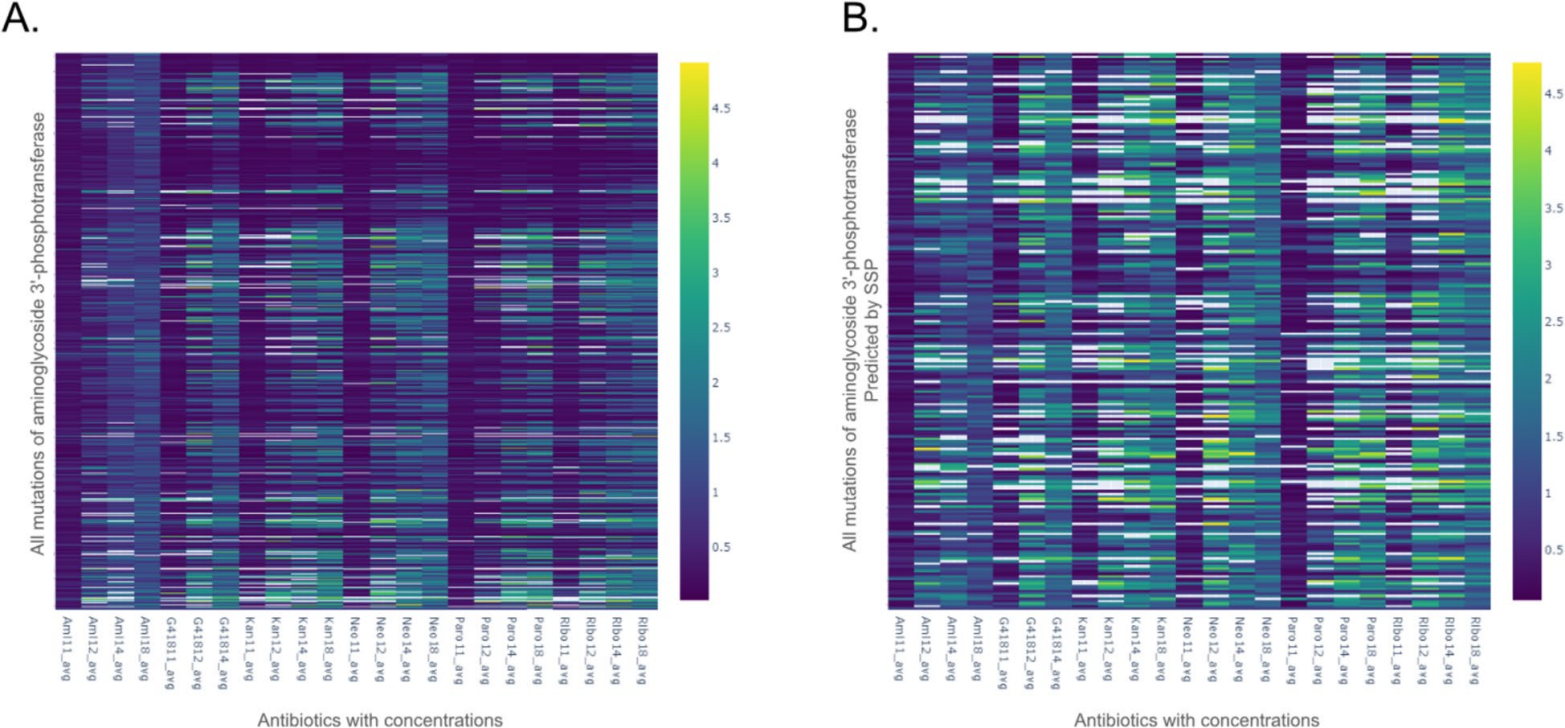
Heatmap visualisations comparing the enrichment values for mutations of aminoglycoside 3'-phosphotransferase. **A** A heatmap representing the entire mutational space of aminoglycoside 3'-phosphotransferase. **B** A heatmap representing only the mutations of aminoglycoside 3'-phosphotransferase that were predicted by the SSP. The X-axis represents the antibiotics and their tested concentrations, while the Y-axis represents the relevant mutations of aminoglycoside 3'-phosphotransferase. Details of antibiotic concentrations and individual enrichment values can be found in SI 1. Each rectangle on the plot indicates the enrichment value for a mutation when exposed to the effects of the specific antibiotic concentration. The Viridis colour map is used to maintain perceptual uniformity. A value of 1 (dark blue) represents no change in enrichment from the wild type, anything below 1 (purple) represents a negative effect on enrichment, while anything above 1 (light blue to yellow) represents a positive enriching effect of the mutation. This figure contrasts the effects of random mutations on the activity of aminoglycoside 3'-phosphotransferase, against the effect of SSP suggested mutations for the same protein. The perceptual increase in ‘brightness’ of **B** over **A** illustrates an increase in the positive impact of mutations on the activity of aminoglycoside 3'-phosphotransferase

Aminoglycoside 3ʹ-phosphotransferase is a protein that confers resistance to aminoglycosides, such as kanamycin, neomycin, paromomycin, ribostamycin, butirosin, and gentamicin B. Melnokov et al. [36] conducted site saturation mutagenesis on this protein, transformed variants into cells and exposed them to six different antibiotics at up to four different concentrations. The amino acid enrichment (the number of identified variants with the particular mutation) was then noted in each case. A value of ~ 1 applies to wild types, while a higher value means more resistance and hence more significant enrichment of that mutant, and vice versa for a value below 1 (Fig. 4).

The average of all enrichment values across antibiotics and their concentrations in the complete dataset (AAC value) was 0.82. This means that a random, single-amino-acid variant is less likely to be resistant than the wild type, and, therefore, will have lower activity. The AAC value of 0.82 may be assumed as a proxy value for random mutations, while 1 is the default value for the wild type. Thus, random single-point mutations are likely to reduce the protein's fitness. SSP generated 221 predictions, all with experimental validation points available from this large-scale site saturation mutagenesis study (Fig. 4). For mutants generated by SSP, the AAC value is 1.36, showing a preferable selection of enriched (more active) variants, thus an increase in fitness if a mutation is selected from SSP’s output. Moreover, 61 of the 221 mutations were predicted at the MAP level, and their AAC value is 1.4. The remaining 160 predictions were made at the SAP level, and their AAC value is 1.34. As the AAC value for MAP level predictions is slightly higher than that for SAP level (1.4 *versus* 1.34), it hints at the possibility that MAPs may be slightly more reliable. This is summarised in Fig. 3C. The comparison of experimentally determined and predicted values are available in the supplementary table SI 1.

### Evolutionary selection

Structure Integrated with Positive Selection (SIPS) is an online resource with positively selected sites mapped onto protein structures from an evolutionary perspective [19]. ADP-ribosylarginine hydrolase, which is one of the example cases of SIPS, has eight positively selected sites with an adjusted p-value threshold of 0.2 or higher. SSP predictions were made for ADP-ribosylarginine hydrolase to see how many of the predictions could be made for positively selected sites. Here, the emphasis was on sites and not the mutation itself, as SIPS only lists sites of evolutionary interest and not what they would mutate into. Out of the eight sites, seven were predicted by SSP, and six were MAPs, implying that SSP can selectively make predictions for sites with evolutionary significance (Table 5).

**Table 5:** Cross-matching positively selected site data of ADP-ribosylarginine hydrolase from SIPS with SSP predictions [19]

Sites selected by SSP	Adjusted p-value	Prediction agreement type
K72	0.0848	Mutation agreeing
P74	0.0473	Mutation agreeing
T77	0.1731	Mutation agreeing
Q78	0.1101	Mutation agreeing
Q109	0.0796	Mutation agreeing
H145	0.05	Non agreeing
L189	0.0002	Mutation agreeing
I355	0.0128	*Not predicted*

## Discussion and conclusions

SSP is a protein design method that employs the prediction of the evolution of amino acids in a protein sequence. It builds a statistical, ancestral sequence reconstruction-guided evolutionary history of a protein sequence [39], which is utilised to extrapolate the possible *future* substitution at a given position. SSP makes these predictions in the context of AAindex scoring [12] applied to the reconstructed evolutionary history for each position in a protein sequence. The AAindices used for SSP have been manually selected to reflect a variety of possibly relevant physiochemical descriptors. The selected set of AAindices can be easily adjusted based on the physico-chemical properties expected to be involved in shaping the evolution of a particular protein.

It should be noted that while SSP utilises ASR, they are both *fundamentally different* techniques with distinct goals. ASR aims to ‘look back’ into the evolutionary history of a protein sequence, while SSP is designed to extrapolate into the potential future of a protein sequence. ASR is generally used for evolutionary analysis [40] and protein engineering [39]. While ancestral proteins are more robust and with unique substrate specificities [32, 41, 42], the engineering scope of ASR is generally along the lines of improving the thermostability of a protein and its expression yield. This is because ancestral proteins, when resurrected, tend to be more robust [43]. SSP can map out potential future evolutionary trajectories of a protein, and it can also be used to engineer proteins.

Proseeker is another tool that simulates natural selection and thus mimics evolution in silico. It uses physicochemical characteristics and structural information to pick sequences that have higher probabilities of evolving a desired function [11]. However, the technique was designed specifically for binding proteins and lacks general applicability. Instead of complete protein sequences, it uses small peptide sequences (13 amino acids), and it also does not filter or select specific AAindices, rather it uses all available AAindices [12]. The selection of relevant indices and then estimating their utility for any tool in this domain is crucial as many indices are redundant, e.g., nine indices for the hydrophobicity: ARGP820101, GOLD730101, JOND750101, PRAM900101, ZIMJ680101, PONP930101, WOLR790101, ENGD860101, and FASG890101 [12]. This can lead to index weighting issues, where a certain physiochemical descriptor may have an exaggerated effect on the outcome. Furthermore, many indices are context-specific, such as hydrophobicity coefficients in specific solutions—from WILM950101 to WILM950104, and weights for alpha-helix at specific window positions—from QIAN880101 to QIAN880139 [12]. Thus a careful selection of indices is a necessary step, SSP used manually curated non-correlated indices (Table 1 and Fig. 1). While the direct comparison between SSP and Proseeker could have been useful, it is hard to achieve as Proseeker works with shortened peptides (13 AA long) instead of the whole protein sequence. Moreover, it specifically requires binding affinity data to score every iteration of in silico evolution, thus making the technique specific to nucleic acid binding peptides. SSP is not limited in terms of the nature or the length of the target protein sequence.

SSP was validated using the datasets from different sources to test for the performance of various properties. In the case of thermostability, SSP made 14 predictions for the cold shock protein CspB, eight of which had a stabilising effect on the protein (ΔΔG < 0), while the remaining six were neutral with ΔΔG values between 0 kcal/mol and 1 kcal/mol. Seven of the predicted mutations also had positive experimentally determined changes in melting temperatures △Tm (°C), including the highest increase in melting temperature of + 16.6 °C, and only three mutation had a negative △Tm (°C) value with the lowest being − 6.9 °C.

SSP was also used to make predictions for aminoglycoside 3'-phosphotransferase [36]. Aminoglycoside 3'-phosphotransferase is an enzyme that confers resistance to aminoglycosides with antibiotic properties. Thus an enhancement of enzyme’s activity can increase the antibiotic resistance of a bacteria that codes for it. SSP made 221 predictions for Aminoglycoside 3'-phosphotransferase with an AAC value of 1.4 at the MAP level, and 1.36 at the SAP level (1 being the value for the wild type, and 0.82 being the average value for random mutagenesis), thus demonstrating predictive prowess in the context of enhancing enzymatic activity, being significantly better than random mutation, while conferring an improvement over the wild type itself.

Validation of mutations predicted from the solubility dataset showed a higher likelihood of a positive or neutral effect on the solubility of the protein, despite the sparseness of the dataset. Furthermore, evolutionary selectivity data for ADP-ribosylarginine hydrolase [19] taken from SIPS and SSP made predictions for 7 of 8 evolutionary selected sites with an adjusted p-value upper threshold of 0.2. This result suggests that SSP is selective in making predictions for sites that tend to evolve under positive selection, thus making a strong case for SSP’s selectivity. However, it should be noted that the size of the dataset is quite small, and more work is required to validate this aspect of the predictor.

Analyzing and validating methods like SSP presents significant challenges. Extracting meaningful insights from diverse datasets with varying experimental standards can be complex due to limited overlap between experimentally observed mutations and the mutations predicted by SSP (Fig. 3). Finding datasets that are not only extensive but also contain experimental data for mutations that coincide with SSP predictions—enabling their validation—proved to be a substantial hurdle. This scarcity necessitated the use of all available validation sets, despite their inherent differences in physicochemical properties. Substantially more mutational data would be needed to have evenly distributed dataset for each protein property.

This study shows that the SSP approach enhances specialised proteins by predicting mutations that improve desired properties, such as thermostability, activity, and solubility. Crucially, it also shows that SSP does not make predictions for sites randomly, but picks sites that are known to evolve under positive selection. In general, SSP method will work better with the proteins under stronger selection evolutionary pressure. Further validation of the predictor with diverse protein structures is desirable to define applicability for protein engineering applications. It should also be noted that the technique has a limitation; it is dependent on the size and quality of the homolog set. The technique will not work if the protein has no or very few homologs. For our pipeline, we suggest having at least 10 trees of 150 protein sequences each, per analysis. However the exact numbers need further exploration.

As the service to the community, we are now integrating SSP as a new module into the easy-to-use web server FireProtASR (https://loschmidt.chemi.muni.cz/fireprotasr/), which will make predictions accessible to non-experts, jointly with related strategies Ancestral Sequence Reconstruction (ASR) and generation of sequences using Variational Autoencoder (VAE) [44].

## Supplementary Information

Supplementary Material 1: Comparison of experimentally determined and predicted values for Aminoglycoside 3'-phosphotransferase, as well as the complete enrichment data.

Supplementary Material 2: A text README file on how to locally run the Successor Sequence Predictor.

Supplementary Material 3: Example case query sequence in FASTA format.

Supplementary Material 4: Example case homolog database in FASTA format.

Supplementary Material 5: Pre-run Ancestral Sequence Reconstruction output.

Supplementary Material 6.

## Data Availability

We provide scripts as a command line application written in Python 3.8, which can be found on GitHub https://github.com/loschmidt/successor-sequence-predictor. We use the LinearRegression module from the scikit-learn library to predict the evolutionary trend in the phylogenetic tree.

## References

[1] Hall BK, Hallgrimsson B (2014) Strickberger’s evolution. Jones And Bartlett.

[2] Gillespie JH (1994) The causes of molecular evolution. Oxford University Press

[3] Kimura M (1985) The neutral theory of molecular evolution. Cambridge University Press

[4] Nosil P, Flaxman SM, Feder JL, Gompert Z (2020) Increasing our ability to predict contemporary evolution. Nat Commun 11(1) 10.1038/s41467-020-19437-x 10.1038/s41467-020-19437-x PMC7645684 33154385

[5] Eyre-Walker A, Keightley PD (2007) The distribution of fitness effects of new mutations. Nat Rev Genet 8(8):610–618. 10.1038/nrg2146 17637733 10.1038/nrg2146

[6] Pál C, Papp B, Lercher MJ (2006) An integrated view of protein evolution. Nat Rev Genet 7(5):337–348. 10.1038/nrg1838 16619049 10.1038/nrg1838

[7] Cano AV, Gitschlag BL, Rozhoňová H, Stoltzfus A, McCandlish DM, Payne JL (2023) Mutation bias and the predictability of evolution. Philos Trans R Soc B 378(1877):20220055. 10.1098/rstb.2022.0055 10.1098/rstb.2022.0055 PMC10067271 37004719

[8] Yang KK, Wu Z, Arnold FH (2019) Machine-learning-guided directed evolution for protein engineering. Nat Methods 16:687–694. 10.1038/s41592-019-0496-6 31308553 10.1038/s41592-019-0496-6

[9] Arnold FH (2019) Innovation by evolution: bringing new chemistry to life (nobel lecture). Angew Chem 58(41):14420–14426. 10.1002/anie.201907729 31433107 10.1002/anie.201907729

[10] Fay JC, Wu CI (2000) Hitchhiking under positive Darwinian selection. Genetics 155(3):1405–1413. 10.1093/genetics/155.3.1405 10880498 10.1093/genetics/155.3.1405 PMC1461156

[11] Raven SA, Payne B, Mitchell AF, Rackham O (2022) In silico evolution of nucleic acid-binding proteins from a nonfunctional scaffold. Nat Chem Biol 18(4):403–411. 10.1038/s41589-022-00967-y 35210620 10.1038/s41589-022-00967-y

[12] Kawashima S (2000) AAindex: amino acid index database. Nucleic Acids Res 28(1):374–374. 10.1093/nar/28.1.374 10592278 10.1093/nar/28.1.374 PMC102411

[13] Livada J, Vargas AM, Martinez CA, Lewis RD (2023) Ancestral sequence reconstruction enhances gene mining efforts for industrial ene reductases by expanding enzyme panels with thermostable catalysts. ACS Catal 13(4):2576–2585. 10.1021/acscatal.2c03859

[14] Musil M, Khan RT, Beier A, Stourac J, Konegger H, Damborsky J, Bednar D (2020) FireProtASR: a web server for fully automated ancestral sequence reconstruction. Brief Bioinform. 10.1093/bib/bbaa337 10.1093/bib/bbaa337 PMC8294521 33346815

[15] Prakinee K, Phaisan S, Kongjaroon S, Chaiyen P (2024) Ancestral sequence reconstruction for designing biocatalysts and investigating their functional mechanisms. JACS Au 4(12):4571–4591. 10.1021/jacsau.4c00653 39735918 10.1021/jacsau.4c00653 PMC11672134

[16] Gumulya Y, Huang W, D’Cunha SA, Richards KE, Thomson RE, Hunter DJ, Baek JM, Harris KL, Boden M, De Voss JJ, Hayes MA (2019) Engineering thermostable CYP2D enzymes for biocatalysis using combinatorial libraries of ancestors for directed evolution (CLADE). ChemCatChem 11(2):841–850. 10.1002/cctc.201801644

[17] Gumulya Y, Baek JM, Wun SJ, Thomson RE, Harris KL, Hunter DJ, Behrendorff JB, Kulig J, Zheng S, Wu X, Wu B (2018) Engineering highly functional thermostable proteins using ancestral sequence reconstruction. Nat Catal 1(11):878–888. 10.1038/s41929-018-0159-5

[18] Brennan CK, Livada J, Martinez CA, Lewis RD (2024) Ancestral sequence reconstruction meets machine learning: ene reductase thermostabilization yields enzymes with improved reactivity profiles. ACS Catal 14(23):17893–17900. 10.1021/acscatal.4c03738

[19] Slodkowicz G, Goldman N (2020) Integrated structural and evolutionary analysis reveals common mechanisms underlying adaptive evolution in mammals. Proc Natl Acad Sci 117(11):5977–5986. 10.1073/pnas.1916786117 32123117 10.1073/pnas.1916786117 PMC7084095

[20] Fasman GD (1989) Practical handbook of biochemistry and molecular biology. CRC Press, New York. 10.1201/9781351072427/handbook-biochemistry-gerald-fasman

[21] Goldsack DE, Chalifoux RC (1973) Contribution of the free energy of mixing of hydrophobic side chains to the stability of the tertiary structure of proteins. J Theor Biol 39(3):645–651. 10.1016/0022-5193(73)90075-1 4354159 10.1016/0022-5193(73)90075-1

[22] Wolfenden RV, Cullis PM, Southgate CCF (1979) Water, protein folding, and the genetic code. Science 206(4418):575–577. 10.1126/science.493962 493962 10.1126/science.493962

[23] Bhaskran R, Ponnuswamy PK (1988) Positional flexibilities of amino acid residues in globular proteins. Int J Pept Protein Res 32(4):241–255. 10.1111/j.1399-3011.1988.tb01258.x 10.1111/j.1399-3011.1984.tb00944.x 6480218

[24] Bull HB, Breese K (1974) Surface tension of amino acid solutions: a hydrophobicity scale of the amino acid residues. Arch Biochem Biophys 161(2):665–670. 10.1016/0003-9861(74)90352-x 4839053 10.1016/0003-9861(74)90352-x

[25] Fauchere J-L, Charton M, Kier LB, Verloop A, Pliska V (2009) Amino acid side chain parameters for correlation studies in biology and pharmacology. Int J Pept Protein Res 32(4):269–278. 10.1111/j.1399-3011.1988.tb01261.x 10.1111/j.1399-3011.1988.tb01261.x 3209351

[26] Zimmerman JM, Eliezer N, Simha R (1968) The characterization of amino acid sequences in proteins by statistical methods. J Theor Biol 21(2):170–201. 10.1016/0022-5193(68)90069-6 5700434 10.1016/0022-5193(68)90069-6

[27] Johnson M, Zaretskaya I, Raytselis Y, Merezhuk Y, McGinnis S, Madden TL (2008) NCBI BLAST: a better web interface. Nucleic Acids Res 36(1):5–9. 10.1093/nar/gkn201 10.1093/nar/gkn201 PMC2447716 18440982

[28] Liu Y, Hayes DN, Nobel A, Marron JS (2008) Statistical significance of clustering for high-dimension, low-sample size data. J Am Stat Assoc 103(483):1281–1293

[29] Sievers F, Higgins DG (2014) Clustal omega. Curr Protoc Bioinformat. 48(1) 10.1002/0471250953.bi0313s48 10.1002/0471250953.bi0313s48 25501942

[30] Stamatakis A (2014) RAxML version 8: a tool for phylogenetic analysis and post-analysis of large phylogenies. Bioinformatics 30(9):1312–1313. 10.1093/bioinformatics/btu033 24451623 10.1093/bioinformatics/btu033 PMC3998144

[31] Tria F, Landan G, Dagan T (2017) Phylogenetic rooting using minimal ancestor deviation. Nat Ecol Evol 1:0193. 10.1038/s41559-017-0193 10.1038/s41559-017-0193 29388565

[32] Hanson-Smith V, Kolaczkowski B, Thornton JW (2010) Robustness of ancestral sequence reconstruction to phylogenetic uncertainty. Mol Biol Evol 27(9):1988–1999. 10.1093/molbev/msq081 20368266 10.1093/molbev/msq081 PMC2922618

[33] sklearn.linear_model.LinearRegression (2023) Scikit-Learn. https://scikit-learn.org/stable/modules/generated/sklearn.linear_model.LinearRegression.html

[34] Klesmith JR, Bacik J-P, Wrenbeck EE, Michalczyk R, Whitehead TA (2017) Trade-offs between enzyme fitness and solubility illuminated by deep mutational scanning. Proc Natl Acad Sci 114(9):2265–2270. 10.1073/pnas.1614437114 28196882 10.1073/pnas.1614437114 PMC5338495

[35] Gribenko AV, Makhatadze GI (2007) Role of the charge-charge interactions in defining stability and halophilicity of the CspB proteins. J Mol Biol 366(3):842–856. 10.1016/j.jmb.2006.11.061 17188709 10.1016/j.jmb.2006.11.061

[36] Melnikov A, Rogov P, Wang L, Gnirke A, Mikkelsen TS (2014) Comprehensive mutational scanning of a kinase *in vivo* reveals substrate-dependent fitness landscapes. Nucleic Acids Res 42(14):e112–e112. 10.1093/nar/gku511 24914046 10.1093/nar/gku511 PMC4132701

[37] Velecký J, Hamsikova M, Stourac J, Musil M, Damborsky J, Bednar D, Mazurenko S (2022) SoluProtMutDB: a manually curated database of protein solubility changes upon mutations. Comput Struct Biotechnol J 20:6339–6347. 10.1016/j.csbj.2022.11.009 36420168 10.1016/j.csbj.2022.11.009 PMC9678803

[38] Stourac J, Dubrava J, Musil M, Horackova J, Damborsky J, Mazurenko S, Bednar D (2020) FireProtDB: database of manually curated protein stability data. Nucleic Acids Res 49(D1):D319–D324. 10.1093/nar/gkaa981 10.1093/nar/gkaa981 PMC7778887 33166383

[39] Spence MA, Kaczmarski JA, Saunders JW, Jackson CJ (2021) Ancestral sequence reconstruction for protein engineers. Curr Opin Struct Biol 69:131–141. 10.1016/j.sbi.2021.04.001 34023793 10.1016/j.sbi.2021.04.001

[40] Cai W, Pei J, Grishin NV (2004) Reconstruction of ancestral protein sequences and its applications. BMC Evol Biol 4:1–23. 10.1186/1471-2148-4-33 15377393 10.1186/1471-2148-4-33 PMC522809

[41] Babkova P, Sebestova E, Brezovsky J, Chaloupkova R, Damborsky J (2017) Ancestral haloalkane dehalogenases show robustness and unique substrate specificity. ChemBioChem 18(14):1448–1456. 10.1002/cbic.201700197 28419658 10.1002/cbic.201700197

[42] Risso VA, Sanchez-Ruiz JM (2017) Resurrected ancestral proteins as scaffolds for protein engineering. In: Directed enzyme evolution: advances and applications. pp. 229–255. 10.1007/978-3-319-50413-1_9

[43] Thomson RE, Carrera-Pacheco SE, Gillam EM (2022) Engineering functional thermostable proteins using ancestral sequence reconstruction. J Biol Chem 298(10):102435. 10.1016/j.jbc.2022.102435 36041629 10.1016/j.jbc.2022.102435 PMC9525910

[44] Kohout P, Vasina M, Majerova M, Novakova V, Damborsky J, Bednar D, Marek M, Prokop Z, Mazurenko S (2025). Engineering Dehalogenase Enzymes Using Variational Autoencoder-Generated Latent Spaces and Microfluidics. JACS Au. 10.1021/jacsau.4c01101. 10.1021/jacsau.4c01101 PMC11862945 40017771

